# The association between the neutrophil-to-lymphocyte ratio and mortality in critical illness: an observational cohort study

**DOI:** 10.1186/s13054-014-0731-6

**Published:** 2015-01-19

**Authors:** Justin D Salciccioli, Dominic C Marshall, Marco AF Pimentel, Mauro D Santos, Tom Pollard, Leo Anthony Celi, Joseph Shalhoub

**Affiliations:** Department of Medicine, Imperial College London, London, SW7 2AZ UK; Institute of Biomedical Engineering, Department of Engineering Science, University of Oxford, Old Road Campus Research Building, Headington, Oxford, OX3 7DQ UK; University College London, Mullard Space Science Laboratory, Gower Street, London, WC1E 6BT UK; Department of Anaesthetics and Critical Care, University College Hospital, Podium 3, Maple Link corridor, University College Hospital, 235 Euston Road, London, NW1 2BU UK; Institute for Medical Engineering & Science, Massachusetts Institute of Technology, 77 Massachusetts Avenue, Cambridge, 02139 MA USA; Division of Pulmonary, Critical Care and Sleep Medicine, Department of Medicine, Beth Israel Deaconess Medical Center, 330 Brookline Avenue, Boston, 02215 MA USA; Division of Vascular Surgery, Department of Surgery and Cancer, Imperial College London, 4 North, Charing Cross Hospital, Fulham Palace Road, London, W6 8RF UK

## Abstract

**Introduction:**

The neutrophil-to-lymphocyte ratio (NLR) is a biological marker that has been shown to be associated with outcomes in patients with a number of different malignancies. The objective of this study was to assess the relationship between NLR and mortality in a population of adult critically ill patients.

**Methods:**

We performed an observational cohort study of unselected intensive care unit (ICU) patients based on records in a large clinical database. We computed individual patient NLR and categorized patients by quartile of this ratio. The association of NLR quartiles and 28-day mortality was assessed using multivariable logistic regression. Secondary outcomes included mortality in the ICU, in-hospital mortality and 1-year mortality. An *a priori* subgroup analysis of patients with versus without sepsis was performed to assess any differences in the relationship between the NLR and outcomes in these cohorts.

**Results:**

A total of 5,056 patients were included. Their 28-day mortality rate was 19%. The median age of the cohort was 65 years, and 47% were female. The median NLR for the entire cohort was 8.9 (interquartile range, 4.99 to 16.21). Following multivariable adjustments, there was a stepwise increase in mortality with increasing quartiles of NLR (first quartile: reference category; second quartile odds ratio (OR) = 1.32; 95% confidence interval (CI), 1.03 to 1.71; third quartile OR = 1.43; 95% CI, 1.12 to 1.83; 4th quartile OR = 1.71; 95% CI, 1.35 to 2.16). A similar stepwise relationship was identified in the subgroup of patients who presented without sepsis. The NLR was not associated with 28-day mortality in patients with sepsis. Increasing quartile of NLR was statistically significantly associated with secondary outcome.

**Conclusion:**

The NLR is associated with outcomes in unselected critically ill patients. In patients with sepsis, there was no statistically significant relationship between NLR and mortality. Further investigation is required to increase understanding of the pathophysiology of this relationship and to validate these findings with data collected prospectively.

**Electronic supplementary material:**

The online version of this article (doi:10.1186/s13054-014-0731-6) contains supplementary material, which is available to authorized users.

## Introduction

More than 5 million patients are admitted to intensive care units (ICUs) each year in the United States with survival rates ranging between 10% to 29% depending on the population studied [1,2]. Systemic inflammation is an integral part of disease processes in critical illness and is commonly associated with the sepsis syndrome [2,3]. Various biomarkers, including acute phase proteins and cytokines, are frequently used in the ICU to assess underlying inflammatory processes in both clinical practice and for research purposes [4-8].

The neutrophil-to-lymphocyte ratio (NLR) is a readily available biomarker that can be calculated based on a complete blood count. NLR has previously been shown to predict outcomes in oncology patients [9] and has been tested in a number of malignancies, including lung [10], ovary [11] and breast [12]. Preoperative NLR has been shown to be prognostic in patients undergoing colorectal cancer resection [13]. Despite the evidence in various patient populations demonstrating a relationship between NLR and mortality, no previous report has described the relationship between NLR and outcomes in a large population of unselected critically ill patients.

Our objective in the present study was to evaluate whether there is an association between NLR and mortality in a population of adult critically ill patients. Our primary hypothesis was that NLR at ICU admission is associated with mortality in critically ill patients. To test this hypothesis, we performed an observational study using a large clinical database of unselected adult critically ill patients.

## Material and methods

### Data source

We performed an observational study using data collected from the Multiparameter Intelligent Monitoring in Intensive Care (MIMIC II) open source clinical database. MIMIC II was developed and is maintained by the Massachusetts Institute of Technology (MIT), Philips Healthcare and Beth Israel Deaconess Medical Center (BIDMC) [14]. Patients included in this dataset were hospitalized between January 2001 and December 2008. The database includes all physiological data recorded in the ICU, clinical variables, results of investigations (including laboratory tests) and survival outcome data. Survival data are obtained postdischarge from the Social Security death records.

The MIMIC II database has received ethical approval from the institutional review boards (IRBs) at BIDMC and MIT, and, because the database does not contain protected health information, a waiver of the requirement for informed consent was included in the IRB approval.

### Patient population

The criteria for inclusion in this study were that the patients had to (1) be adults (>17 years of age) at ICU admission, regardless of admitting diagnosis; and had to (2) have neutrophil and lymphocyte counts measured at ICU admission. The exclusion criteria were (1) missing neutrophil and lymphocyte data at ICU admission, (2) missing covariate data for multivariable adjustments and (3) repeat admissions to the ICU. Patients who met the inclusion criteria and none of the exclusion criteria were included in the final cohort for investigation.

From each patient record, we extracted the following available variables from the database at ICU admission: demographic data, including age in years, sex, comorbid conditions as coded and defined in the International Classification of Diseases, Ninth Revision (ICD-9); vital statistics data and laboratory data; and admission severity of illness scores (Simplified Acute Physiology Score (SAPS I) and Sequential Organ Failure Assessment (SOFA)). SAPS I and SOFA scores were computed automatically in the database as previously described [15,16].

### Predictor and outcome variables

The primary exposure of interest was NLR measured at ICU admission. NLR was computed based on ICU admission laboratory data as a ratio of neutrophil/lymphocyte values, which are recorded in the complete blood count, and patients were categorized by quartile of baseline NLR value.

The primary outcome was 28-day mortality. Secondary outcomes were mortality in the ICU, in-hospital mortality and 1-year mortality. Mortality data were collected from the Social Security death records.

### Statistical analysis and modeling strategy

Data for continuous variables are presented as median with interquartile range (IQR). Dichotomous and categorical variables are presented as frequencies with percentages. We assessed the distribution of the primary predictor variable graphically using histograms. We computed the median with IQR for the primary predictor variable and categorized patients according to quartile of baseline NLR at ICU admission, with the first quartile treated as the reference group for all subsequent analyses.

The following variables were considered for multivariable adjustments: age, sex, baseline laboratory data (white cell count, neutrophil and lymphocyte data, hemoglobin, red blood cell count and hematocrit, creatinine and blood urea nitrogen, and electrolyte data), baseline vital statistics data (heart rate, respiratory rate, systolic blood pressure, Glasgow Coma Scale score), comorbid diseases (congestive heart failure, chronic obstructive pulmonary disease, cardiac arrhythmia, valvular disease, peripheral vascular disease, hypertension, hypothyroidism, metastatic disease or lymphoma, liver failure or renal failure) and baseline clinical severity markers (SAPS I and SOFA scores).

We assessed the association of quartile of NLR with primary and secondary outcomes using univariate logistic regression, and we report the odds ratios (ORs) and 95% confidence intervals (CIs). To assess the association of quartiles of NLR, we attempted to adjust for potential confounding with multivariable logistic regression. We initially included in the multivariable model all variables with a statistically significant univariate association with outcome to control for potentially clinically relevant confounding variables. To avoid overfitting, we manually removed from the model variables that were not associated with the outcome and used the Akaike Information Criterion to define the final model. We report ORs and 95% CIs for the final model. To account for potential clustering within ICUs, we used a generalized estimating equation with a variance-covariance structure with compound symmetry. We generated Kaplan-Meier curves to assess the probability of survival across quartiles of NLR.

### Subset and sensitivity analyses

We conducted two confirmatory analyses to further the primary hypothesis. First, as patients presenting to the ICU with sepsis are likely to have marked neutrophilia, which could significantly influence the overall association between NLR and outcomes, we planned *a priori* to perform a subgroup analysis of patients with sepsis upon presentation compared with patients without sepsis. We defined the subgroup of patients with sepsis according to the criteria outlined by Angus *et al.* [2]. Patients were identified by using ICD-9 codes for diagnosis of bacterial and fungal infectious process as well as for a diagnosis of acute organ dysfunction as recorded in the MIMIC II database. Using multivariable regression, we assessed the relationship between NLR and outcome in the cohorts of patients with and without sepsis. We also performed a *post hoc* test of the sepsis cohort after removing patients with sepsis who were identified as having neutropenia (<1.5 × 10^9^ cells/L) at the time of presentation to the ICU.

Second, a number of patients were not included in the primary analysis because they were missing NLR data at the time of admission to the ICU. For this reason, we performed a *post hoc* sensitivity analysis using the first neutrophil and lymphocyte data recorded in the ICU. Whereas patients included in the primary analysis had NLR data measured upon admission to the ICU, we assessed the first recorded NLR value in this sensitivity analysis, regardless of its relation to the time of admission to the ICU. We repeated the univariate and multivariable analyses to assess 28-day mortality across quartiles of NLR, and we report the adjusted ORs and 95% CIs. In addition, to assess the possibility of a selection bias, we tested, using the χ^2^ test, the difference in the crude 28-day survival between patients with and without NLR measured in the ICU.

### Reclassification of severity scores

Model discrimination was assessed by calculation of the receiver operating characteristic (ROC) curves and report the area under the ROC curve (AUC). In addition to ROC analysis, we assessed the effect of adding NLR to risk-adjusted models by using reclassification analytical methods. As described by Pencina *et al*. [17,18], reclassification analysis is a method that has been recommended for assessing the incremental contribution of biomarkers to risk prediction. We calculated the net reclassification improvement (NRI) index, which is used to measure the ability of a new model (with the new biomarker, in this case NLR) to reclassify a high-risk individual as higher risk and a low-risk individual as lower risk. We also assessed the integrated discrimination improvement (IDI) index, which takes into account the overall joint improvement in calculating the sensitivity and specificity of the new model.

All reported tests of the data are two-sided with a significance level of 5%, and CIs are reported as two-sided 95% CIs. Statistical tests of the data were performed in SAS v9.3 software (SAS Institute, Cary, NC, USA) as well as in R v3.0.1 (R Foundation for Statistical Computing, Vienna, Austria).

## Results

### Population characteristics

There were a total of 5,763 patients with complete neutrophil and lymphocyte data available upon admission, and all of these patients had complete data available for the primary outcome. After excluding patients with missing data from any of the selected covariates, 5,056 patients made up the final cohort for analysis.

The median age of the cohort was 65 years (IQR, 51 to 78), and 47% of patients were female. The median SAPS I score was 14 (IQR, 10 to 18), and the median SOFA score was 6 (IQR, 3 to 9). The majority (54%) of the patients were admitted to the ICU from the emergency department setting, with 56% of patients treated as medical inpatients, 20% as surgical admissions and an additional 18% treated in the cardiac care unit. Additional baseline characteristics of the study population are shown in Table 1.

**Table 1:** **Baseline characteristics of the study population** ^**a**^

**Parameter**		**Total cohort (** ***N*** **= 5,056)**	**Sepsis subgroup (** ***n*** **= 1,832)**
Age, yr		65 (51 to 78)	69 (55 to 80)
Female sex, *n* (%)		2,377 (47)	879 (48)
Care unit, *n* (%)			
	Cardiac intensive care unit	1,264 (25)	384 (21)
	Cardiac surgical intensive care unit	1,154 (23)	427 (23)
	Medical intensive care unit	2,361 (47)	979 (53)
	Surgical intensive care unit	227 (6)	42 (2)
Comorbid conditions, *n* (%)			
	Congestive heart failure	1,254 (25)	651 (36)
	Chronic obstructive pulmonary disease	953 (19)	343 (19)
	Diabetes	1,167 (23)	474 (26)
	Hypertension	1,518 (30)	499 (27)
Mechanical ventilation (first 24 hr), *n* (%)		2,343 (46)	934 (51)
Vasopressor use (first 24 hr), *n* (%)		1,397 (28)	756 (41)
Glasgow Coma Scale score (IQR)		14 (8 to 15)	14 (8 to 15)
Simplified Acute Physiology Score I (IQR)		14 (10 to 18)	16 (12 to 20)
Sequential Organ Failure Assessment score (IQR)		6 (3 to 9)	8 (4 to 11)

^a^Data are median and interquartile range (IQR) or number and percentage.

The median NLR for the entire cohort was 8.9 (IQR, 4.99 to 16.21). Selected hematologic laboratory data across quartiles of NLR are provided in Table 2. A total of 966 (19%) of the patients died by 28 days, the primary outcome in this study. The overall in-hospital mortality rate was 17%, and 31% of patients had died at 1-year following ICU admission.

**Table 2:** **Laboratory data across quartiles of neutrophil-to-lymphocyte ratio**

	**Neutrophil-to-lymphocyte ratio quartile (range)**			
**Parameter**	**First quartile**	**Second quartile**	**Third quartile**	**Fourth quartile**
	**(<4.99)**	**(4.99 to 8.90)**	**(8. 90 to 16.21)**	**(>16.21)**
White blood cell count, ×10^9^ cells/L	8.4 (5.6 to 11.4)	11.3 (8.7 to 14.7)	13.4 (10.0 to 18.1)	16.2 (11.6 to 22.1)
Red blood cell count, ×10^12^ cells/L	3.64 (3.11 to 4.22)	3.72 (3.25 to 4.25)	3.69 (3.27 to 4.21)	3.69 (3.25 to 4.17)
Hematocrit, %	32.5 (28.1 to 37.4)	33.1 (28.8 to 37.7)	33.0 (29.3 to 37.2)	33.1 (29.1 to 37.1)
Hemoglobin, g/dl	11.0 (9.5 to 12.9)	11.2 (9.7 to 12.8)	11.1 (9.8 to 12.6)	11.0 (9.8 to 12.5)
Mean red cell volume, fl	90.0 (86.0 to 94.0)	89.0 (85.0 to 93.0)	89.0 (85.0 to 93.0)	90.0 (86.0 to 94.0)
Red cell distribution width	14.4 (13.5 to 15.9)	14.4 (13.4 to 15.9)	14.4 (13.5 to 15.9)	14.8 (13.7 to 16.3)

### Association of neutrophil-to-lymphocyte ratio with primary and secondary outcomes

**Figure 1:**
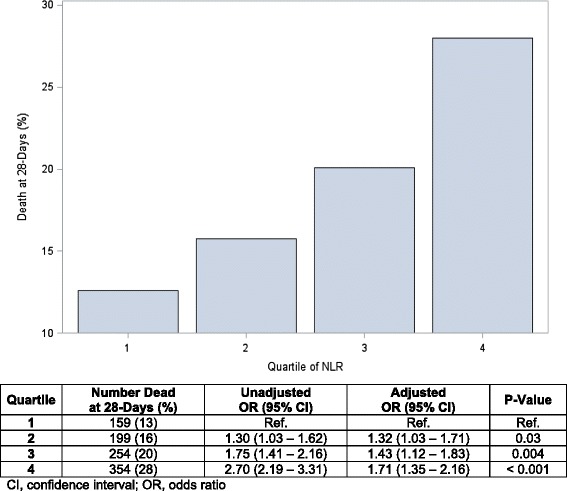
**Twenty-eight-day mortality rates across quartiles of neutrophil-to-lymphocyte ratio (unadjusted and adjusted results).** Crude mortality rates with percentages for patients whose neutrophil-to-lymphocyte ratio (NLR) was measured at the time of admission to the ICU shows a stepwise increase in mortality with increasing quartile of NLR (first quartile = 13%, second quartile = 16%, third quartile = 20%, fourth quartile = 28%). Results of unadjusted and adjusted analyses for quartile of NLR and mortality with Odds Ratios (ORs) and 95% Confidence Intervals (CIs) are also provided. First Quartile < 4.99, second Quartile = 4.99 – 8.90, third Quartile = 8.90 – 16.21, fourth Quartile > 16.21.

**Figure 2:**
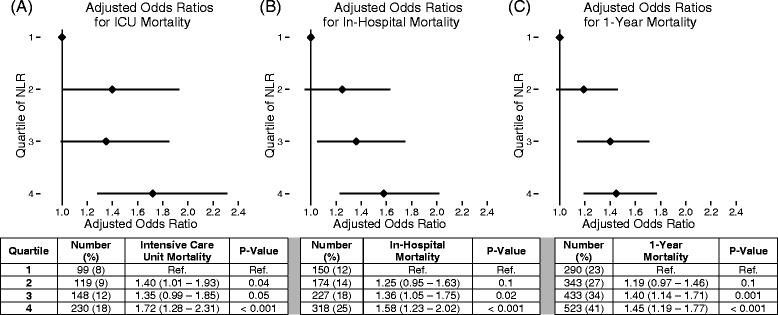
**Multivariable analysis of secondary outcomes.** After multivariable adjustments, we found a stepwise increase in mortality across all secondary outcomes, including intensive care unit (ICU) mortality **(A)**, in-hospital mortality **(B)** and 1-year mortality **(C)**. Point estimates represent the odds ratios (ORs), and error bars represent the 95% confidence intervals (CIs). Numeric ORs and 95% CIs are provided for each of the secondary outcomes. NLR, Neutrophil-to-lymphocyte ratio.

**Figure 3:**
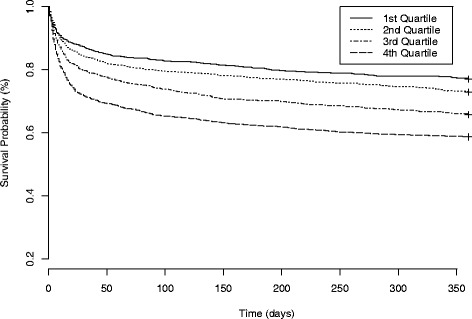
**Kaplan-Meier survival analysis plot for 1-year mortality with quartiles of neutrophil-to-lymphocyte ratio.** The curves demonstrate that patients can be stratified for long-term survival on the basis of neutrophil-to-lymphocyte ratio (NLR) at the time of presentation to the intensive care unit. Patients in the first NLR quartile had the highest probability of survival at 1 year, and patients in the fourth NLR quartile had the lowest probability of survival at 1 year.

There was a statistically significant stepwise increase in crude mortality rate with increasing quartile of baseline NLR. The crude mortality rates by increasing quartile of NLR were as follows: first quartile = 159 (13%), second quartile = 199 (16%), third quartile = 254 (20%) and fourth quartile 354 (28%) (*P* < 0.001). In unadjusted analysis, we found a statistically significant relationship between increasing quartile of NLR and 28-day mortality. This stepwise increase in risk of death remained statistically significant after multivariable adjustments, and similar trends in mortality were present for all secondary outcomes tested (Figures 1 and 2, respectively). The relationship between NLR and 1-year mortality is shown in the Kaplan-Meier curves in Figure 3.

### Subset and sensitivity analyses

Compared with patients without sepsis (*n* = 3,224), patients admitted to the ICU with sepsis (*n* = 1,832) had a statistically significantly higher baseline NLR value at the time of admission (10.9 (IQR, 5.8 to 21) vs. 8.1 (IQR, 4.6 to 14.3); *P* < 0.001). For patients without sepsis, there was a statistically significant stepwise increase in the risk of death at 28 days with increasing quartile of NLR in both univariate and multivariable analyses (adjusted ORs: first quartile = reference value; second quartile = 1.18 (95% CI, 0.81 to 1.72; *P* = 0.4); third quartile = 1.69 (95% CI, 1.20 to 2.40); *P* = 0.003); fourth quartile = 2.13 (95% CI, 1.52 to 2.99); *P* < 0.001). In patients who met the sepsis criteria at ICU admission, there was no relationship between NLR and 28-day mortality (adjusted ORs: first quartile = reference value; second quartile = 1.11 (95% CI, 0.79 to 1.57); third quartile = 0.92 (95% CI, 0.66 to 1.30); fourth quartile = 1.23 (95% CI, 0.86 to 1.70); all *P* > 0.05). After removing the patients with sepsis and neutropenia, there was no relationship between NLR and 28-day mortality (adjusted ORs: first quartile = reference value; second quartile = 1.23 (95% CI, 0.86 to 1.74); third quartile = 1.04 (95% CI, 0.73 to 1.47); fourth quartile = 1.32 (95% CI, 0.94 to 1.85); all *P* > 0.05).

A total of 1,942 patients who did not have baseline NLR values were assessed in the *post hoc* analysis of the first measured NLR values and outcomes. The crude mortality rate across quartiles of NLR were as follows: first quartile = 13%, second quartile = 21%, third quartile = 21%, fourth quartile = 30% (*P* < 0.001). After multivariable adjustments, there was a significant trend for increasing mortality with increasing quartile of NLR; however, this did not follow a stepwise pattern as it did in the primary analysis (adjusted ORs: first quartile = reference value; second quartile = 1.55 (95% CI, 1.08 to 2.23), *P* = 0.02); third quartile = 1.30 (95% CI, 0.90 to 1.86), *P* = 0.2); fourth quartile = 1.99 (95% CI, 1.41 to 2.82), *P* < 0.001).

### Effect of neutrophil-to-lymphocyte ratio on discrimination and reclassification

When we took into consideration the primary outcome of 28-day mortality in the entire study cohort, the addition of NLR to SAPS I score improved the AUC from 0.746 to 0.757 (*P* < 0.001). Reclassification statistics showed a significant improvement in NRI index of 0.032 (*P* < 0.001), indicating that, on average, 3.2% of patients had their 28-day mortality predictions from SAPS I score accurately reclassified with the addition of NLR. Similarly, the IDI index was 0.008 (*P* < 0.001), indicating that an aggregate measure of sensitivity and specificity was superior for SAPS I score and NLR compared with SAPS I score only. The results for the nonseptic and septic cohorts were similar (Additional file 1: Table S1).

## Discussion

We found that the NLR measured at the time of admission to ICU was associated with 28-day mortality in a population of unselected critically ill patients. NLR was able to accurately stratify patients in terms of both short-term and long-term mortality. Whereas NLR remained statistically significantly associated with outcomes in patients without sepsis, there was no relationship between NLR and outcomes in patients with sepsis. These findings remained robust after adjustment for multiple potential confounding variables, suggesting that NLR may be independently associated with outcomes in critical illness.

The hypothesis that NLR is associated with outcomes is based primarily on the physiological link between neutrophilia and lymphopenia with systemic inflammation and stress. First reported by Zahorec *et al*. [9], the NLR may be indicative of the patient’s response to inflammatory insult, with neutrophils rising in response to stress, which, when overwhelming, induces lymphocyte apoptosis [19-21]. For example, Heffernan *et al.* identified the presence of concurrent lymphopenia and neutrophilia in trauma patients and patients who met the criteria for the systemic inflammatory response syndrome [22]. Lymphocytes are important for the regulation of an appropriate inflammatory response, and their loss due to apoptosis, cellular exhaustion and downregulation may perpetuate a detrimental inflammatory state [22,23]. Taken together, the resulting increase in NLR may identify patients who have less physiological reserve to survive the inflammatory insult and concomitant decreased survival rates.

Previous reports have highlighted the association between increased NLR and worse outcomes in patients with cancer of the pancreas [24,25], breast [12], lung [10] and colon [13]. In addition to cohorts of oncology patients, in a prospective study, Suliman *et al.* found that a higher NLR was associated with higher rates of mortality in patients admitted with acute coronary syndrome [26]. NLR has been investigated for its association with adverse outcomes in acute pancreatitis and has been identified as a significant predictor of ICU admission and a longer stay [27]. These data suggest that NLR is important in multiple patient populations and further support our hypothesis that NLR reflects the severity of the underlying systemic disturbance. Compared with other investigations of NLR in non-critically ill populations, our investigation revealed a significantly higher median NLR. This is consistent with the proposed mechanism of NLR representing elevated systemic inflammation and severity of illness seen in the ICU.

We assessed the relationship between NLR and outcome in patients with sepsis as a subgroup because immune dysregulation is intrinsic to the disease process. Although we found an association between NLR and outcome in the nonseptic cohort, there was no relationship between NLR and outcome in the subgroup of patients with sepsis at the time of admission to the ICU. These findings are in contrast to those of a previous study of NLR tested in a population of oncology patients with sepsis [9] and previous findings that have demonstrated an association between lymphopenia and the sepsis syndrome [28]. Additionally, previous studies have demonstrated an association between neutrophilia and mortality in patients with sepsis [29,30]. In contrast, Bermejo-Martín *et al*. found an association between low circulating neutrophil count and mortality [31]. It is hypothesized that patients with sepsis who have low circulating neutrophil counts may have difficulty mounting an appropriate innate immune response. In addition, the sepsis syndrome may increase neutrophil adhesion to the vascular endothelium (and a resultant decrease in measured levels of circulating neutrophils), causing endothelial damage, a common complication of the sepsis syndrome [31]. Further, it is possible that neutrophils can exist in the circulation in varying functional states, and a cross-sectional assessment (as in the present study) relating to the neutrophil or lymphocyte count may be inadequate to understand the effect of these parameters on diseases such as the sepsis syndrome that last days to weeks [32].

The strengths of this study include a large population size and an unselected group of patients, which provided increased generalizability in ICU patients and external validity of our findings in the critical care setting. We were able to use a large clinical dataset with a number of measured characteristics controlled for with multivariable modeling. Furthermore, although our study was designed to assess NLR in a population of unselected critically ill patients, we planned *a priori* to test any difference in the relationship between patients with and without sepsis. This sensitivity analysis was planned in advance, as the pathophysiology of the sepsis response in the critical care setting varies widely, and it is likely that patients with sepsis are inherently different from other critically ill patient populations. Given the results of this sensitivity analysis, it is important for future investigations of NLR in critically ill populations to consider and assess accordingly any differences between patient subpopulations.

This study has a number of limitations that should be taken into consideration when interpreting the results. We conducted a single-center observational study, and thus, as with any observational study, the potential remains for residual confounding. Further associations identified are dependent upon covariates in the model, and other variables not recorded in the database may affect the results if included in an alternative model. We assessed the difference in association between NLR and outcomes in patients who presented to the ICU with sepsis compared with those without sepsis and categorized patients using the Angus *et al*. criteria [2]. However, sepsis is inherently difficult to classify, and misclassification could have inappropriately shifted our results away from or toward the null set.

In addition, we attempted to assess the incremental effect of adding NLR to an existing severity of illness scoring system (SAPS I). However, additional severity of illness scores have been derived since the development of SAPS I, and this may limit our conclusion about the incremental improvement of NLR over existing severity of illness scores. A number of patients in this investigation did not have their neutrophil or lymphocyte data recorded, and we are unable to assess the reason for this using this dataset. It is possible that these data were reported only at the request of the treating clinical team and therefore that an additional selection bias may have been introduced into the current analysis. In future investigations that are designed prospectively to measure NLR data of sequential patients, researchers may be better able to assess the utility of reporting neutrophil and lymphocyte data on all patients presenting to the ICU.

## Conclusions

The NLR measured upon admission to the ICU was associated with both short- and long-term mortality in adult critically ill patients. This relationship was strongest in patients without sepsis. NLR may be a useful indicator of the inflammatory response in adult critical illness. The mechanisms underlying these associations are yet to be fully elucidated and should be the focus of future prospective clinical research.

## Key messages

- NLR was associated with outcomes in various populations and may represent underlying inflammation.
- NLR was associated with mortality in a cohort of adult critically ill patients.
- The relationship between NLR and mortality was strongest in patients who did not have evidence of sepsis.
- These findings should be validated prospectively in further research.

## Additional file

Additional file 1: Table S1. Results of reclassification indices for NLR with SAPS-I scoring.

## Abbreviations

BIDMC: Beth Israel Deaconess Medical Center
CI: Confidence interval
ICD-9: International Classification of Diseases, Ninth Revision
ICU: Intensive care unit
IDI: Integrated Discrimination Improvement
IRB: Institutional review board
MIMIC II: Multiparameter Intelligent Monitoring in Intensive Care II research database
MIT: Massachusetts Institute of Technology
NLR: Neutrophil-to-lymphocyte ratio
OR: Odds ratio
SAPS I: Simplified Acute Physiology Score
SOFA: Sequential Organ Failure Assessment
