# Supplementary material for: The association between the neutrophil-to-lymphocyte ratio and mortality in critical illness: an observational cohort study
Source: Crit Care. 2015 Jan 19;19(1):13. doi: 10.1186/s13054-014-0731-6 (PMC4344736; doi:10.1186/s13054-014-0731-6)
Supplement: Additional file 1: Table S1. — Results of reclassification indices for NLR with SAPS-I scoring. [file 13054_2014_731_MOESM1_ESM.docx]

**Additional file 1:** **Table S1: Results of reclassification indices for NLR with SAPS-I scoring.**

|  | **Study Cohort** | | | **Septic Cohort** | | | **Non-Septic Cohort** | | |
| --- | --- | --- | --- | --- | --- | --- | --- | --- | --- |
|  | **SAPS** | **SAPS/NLR** | **P-value** | **SAPS** | **SAPS/NLR** | **P-value** | **SAPS** | **SAPS/NLR** | **P-value** |
| **AUC** | 0.746 | 0.757 | < 0.001 | 0.694 | 0.699 | 0.01 | 0.765 | 0.777 | 0.002 |
|  | **SAPS vs. SAPS/NLR** | |  | **SAPS vs. SAPS/NLR** | |  | **SAPS vs. SAPS/NLR** | |  |
| **NRI** | 0.032 | | < 0.001 | 0.037 | | < 0.001 | 0.061 | | < 0.001 |
| **IDI** | 0.008 | | < 0.001 | 0.003 | | 0.06 | 0.013 | | < 0.001 |

AUC, area under the receiver-operating characteristic curve; IDI, integrated discrimination improvement; NLR, Neutrophil-to-Lymphocyte Ratio; NRI, net reclassification improvement; SAPS, Simplified Acute Physiology Score I
